# Addressing the social determinants of health: a case study from the Mitanin (community health worker) programme in India

**DOI:** 10.1093/heapol/czu074

**Published:** 2014-09-11

**Authors:** Sulakshana Nandi, Helen Schneider

**Affiliations:** ^1^Public Health Resource Network(PHRN), 28, New Panchsheel Nagar, Near Katora Talab, Civil Lines, Raipur 492001, India and ^2^School of Public Health, University of the Western Cape, Robert Sobukwe Road, Bellville, South Africa 7535

**Keywords:** Case study method, community health worker, India, Mitanin, nutrition, primary healthcare (PHC) approach, qualitative research, social determinants of health, violence against women

## Abstract

The Mitanin Programme, a government community health worker (CHW) programme, was started in Chhattisgarh State of India in 2002. The CHWs (Mitanins) have consistently adopted roles that go beyond health programme-specific interventions to embrace community mobilization and action on local priorities. The aim of this research was to document how and why the Mitanins have been able to act on the social determinants of health, describing the catalysts and processes involved and the enabling programmatic and organizational factors. A qualitative comparative case study of successful action by Mitanin was conducted in two ‘blocks’, purposefully selected as positive exemplars in two districts of Chhattisgarh. One case focused on malnutrition and the other on gender-based violence. Data collection involved 17 in-depth interviews and 10 group interviews with the full range of stakeholders in both blocks, including community members and programme team. Thematic analysis was done using a broad conceptual framework that was further refined. Action on social determinants involved raising awareness on rights, mobilizing women’s collectives, revitalizing local political structures and social action targeting both the community and government service providers. Through these processes, the Mitanins developed identities as agents of change and advocates for the community, both with respect to local cultural and gender norms and in ensuring accountability of service providers. The factors underpinning successful action on social determinants were identified as the significance of the original intent and vision of the programme, and how this was carried through into all aspects of programme design, the role of the Mitanins and their identification with village women, ongoing training and support, and the relative autonomy of the programme. Although the results are not narrowly generalizable and do not necessarily represent the situation of the Mitanin Programme as a whole, the explanatory framework may provide general lessons for programmes in similar contexts.

**KEY MESSAGES**

- Action on social determinants by Mitanins followed processes of raising awareness and mobilizing collectives of women, engaging and revitalizing local political structures, and social action within the community and vis-à-vis the various government service providers.
- The Mitanin developed identities as ‘agents of change’ and ‘advocates for the community’.
- The original ‘intent’ of the programme, and how this was carried through into all aspects of programme design, the role of the Mitanins and their identification with village women, ongoing training, regular support systems and the relative autonomy of the Mitanin Programme infrastructure were the factors underpinning successful action on social determinants.

## Introduction

The Commission on Social Determinants of Health (CSDH 2008), convened by the World Health Organization (WHO) in 2005, reiterated the need to address the social, economic and political determinants of health alongside healthcare provision. The social determinants of health may be defined as ‘the circumstances in which people are born, grow up, live, work and age, and the systems put in place to deal with illness. These circumstances are in turn shaped by a wider set of forces: economics, social policies, and politics’ (WHO undated).

Community health workers (CHWs) are considered an integral part of the primary healthcare (PHC) approach (Werner and Sanders 1997) and have been a feature of health systems for over 50 years (Lehmann and Sanders 2007). However, the roles and activities of CHWs have varied across different programmes and countries (Lehmann and Sanders 2007). As Lehmann and Sanders (2007, p. 5) write, ‘the early literature emphasizes the role of the village health workers (VHWs), which was the term most commonly used at the time, as not only (and possibly not even primarily) a healthcare provider, but also as an advocate for the community and an agent of social change, functioning as a community mouthpiece to fight against inequities and advocate community rights and needs to government structures: in David Werner’s famous words, the health worker as “liberator” rather than “lackey” (Werner 1981)’.

Unfortunately, there are few studies exploring the empowerment or community mobilization roles of CHWs. One reason could be that there are few CHW programmes that articulate and visualize this role for CHWs. Another factor may be that funding of CHW programmes and their evaluations are compelled to focus on individual health outcomes and thereby ignore work on social determinants (Ingram *et al.* 2008).

However, where these have been explicitly studied, it has been found that the CHWs can play the role of community advocates and ‘change agents, empowering individuals, their community, and themselves’ (Becker *et al.* 2004, p. 340). They can also provide leadership to address a number of social, economic and political determinants of health and social inequity (Farquhar *et al.* 2005; Prasad and Muraleedharan 2007; Ingram *et al.* 2008; Pérez and Martinez 2008). Factors identified as facilitating action on social determinants of health are: selection by community (Werner 1981; Bender and Pitkin 1987; Schulz *et al.* 2002; Lehmann and Sanders 2007); flexible selection criteria (Krishnamurthy and Zaidi 2005); acceptability by the community, level of autonomy, appropriate and adequate training, and on-the job support (Werner 1981; Bender and Pitkin 1987; Schulz *et al.* 2002; Farquhar *et al.* 2005; Krishnamurthy and Zaidi 2005; Ingram *et al.* 2008); regular meetings and non-threatening supervision (Schulz *et al.* 2002; van Ginneken *et al.* 2010); self-perception as leaders (Ingram *et al.* 2008) and accountability to the community rather than to health staff (Bender and Pitkin 1987; Sanders 1990; van Ginneken *et al.* 2010).

Fewer studies have described the processes of action on social determinants by CHWs. Some of the processes described relate to mobilizing community groups to address problems, building knowledge and leadership among CHWs, and opening up dialogue with other stakeholders (Schulz *et al.* 2002; Farquhar *et al.* 2005; Ingram *et al.* 2008).

In 2002, the government of the State of Chhattisgarh, India, initiated a CHW programme called the Mitanin (lifelong friend) Programme. There are nearly 60 000 Mitanins, all women, covering almost all the rural hamlets of the state [State Health Resource Centre (SHRC) 2010]. Although it is a government programme, the SHRC, an autonomous organization, facilitates the implementation of the programme.

The Mitanins are women volunteers whose role is to undertake family level outreach services, community-organization building and social mobilization on health and its determinants along with advocacy for improvement in the health system (SHRC 2003a; Sundararaman 2007). Lessons from this programme led to the formulation of a countrywide CHW programme called the Accredited Social Health Activist (ASHA) Programme under the National Rural Health Mission (2005a, 2005b) and all the Mitanins were subsequently recognized as ASHAs.

Although it is recognized that CHWs in both the ASHA and the Mitanin Programmes have addressed the social determinants of health (Sundararaman 2007; National Health Systems Research Center [NHSRC] 2010), there has been little in-depth documentation of such action. Beyond India, there is also limited research on the experiences of CHW programmes in addressing social determinants of health in developing countries (Lehmann and Sanders 2007).

Responding to an important political opportunity, the Mitanin Programme was created and scaled up over a short period of time. As a result no baseline studies were conducted (Sundararaman 2007), and while playing an influential role in the decision to adopt the national ASHA Programme, the Mitanin Programme has not, in fact, been extensively evaluated with respect to its health impacts. However, over a 10-year period (2000–10), Chhattisgarh’s rural infant mortality rate declined at a greater rate than the rest of India: by 45% (from 95 to 52/1000 live births), compared with India’s overall 31% decline (74 to 51/1000 live births) (Registrar General, India [SRS] 2002, 2011). National Family Health Surveys have also recorded greater rates of improvement in infant breast-feeding and anaemia in women and children relative to the rest of India (Rajshekhar 2010). Although it is not possible to attribute these gains narrowly to the Mitanin Programme, it has been the most significant health intervention in the state, targeting specifically maternal and child health (MCH). A recent quasi-experimental evaluation of a nutrition intervention in Chhattisgarh suggested that the Mitanin Programme was responsible for 4.22% and 5.64% annual average reductions in underweight and stunting, respectively, which were higher than the national averages (Vir *et al.* 2014).

If the outcomes of the Mitanin Programme have not been extensively studied, numerous formative evaluations, routine programme assessments and local media reports have documented the way Mitanins have embraced social and community roles that extend well beyond the classic MCH interventions associated with CHW programmes (Nandi 2005; Batiwalla 2007; Sundararaman 2007; Rajshekhar 2010; European Union State Partnership Programme [EUSPP] 2011; Dasgupta 2012).^1^

This study thus sought to describe the role of the Mitanins in addressing social determinants of health in Chhattisgarh State of India. Its objectives were to map the scope of the Mitanins’ work in Chhattisgarh; describe the processes by which they address social determinants of health, specifically focusing on poor nutrition and violence against women and explore the challenges faced and the facilitating factors in their action in addressing these social determinants of health.

The study was conducted in Durgkondal block of Kanker district and Manendragarh block of Koriya district. Durgkondal is situated 140 km south of the state capital, Raipur, with nearly half of its area under forests. Manendragarh block, situated at the northwest corner of Chhattisgarh, is at a distance of 340 km from the state capital with the district having a forest cover of more than 59%. Both blocks have high proportions of indigenous population.

## Methods

An exploratory comparative case study design using qualitative research methods was adopted for the study. A case was defined as action by a CHW (Mitanin) or team of CHWs (Mitanins) on nutrition or violence against women, which has resulted in a positive change in the particular social determinant of health in the context of the village/cluster of villages for which the CHW/s are responsible.

### Sampling

Sampling of the two particular cases was undertaken ‘to identify the cases that will provide a full and sophisticated understanding of all aspects of the phenomenon’ (Rice and Ezzy 1999, p. 42) and followed the replication logic (Yin 2009, p. 54). The cases in the study were selected purposefully through discussions with the field and management staff of the Mitanin Programme, and other stakeholders as ones with ‘exemplary outcomes in relation to the research question’ (Yin 2009, p. 59).

The cases selected were in two ‘blocks’ (sub-districts): Durgkondal for action on nutrition and Manendragarh for action on violence against women. Blocks were selected as they are the basic unit of Mitanin Programme implementation. The themes of nutrition and violence against women were chosen for study as both malnutrition and gender are critical issues in the Indian context (Ramalingaswami *et al.* 1996; Joe *et al.* 2008); they reflect both structural (gender) and intermediary (nutrition) determinants of health inequity (CSDH 2008) and were identified as the recurring themes in the action by the Mitanins on social determinants of health (Batiwalla 2007; Sundararaman 2007). Sampling of respondents within each case was done through snowball sampling (Rice and Ezzy 1999, p. 45).

### Data collection

Data collection was undertaken through in-depth individual interviews and group interviews using an interview guide based on a broad conceptual framework, with CHWs, community members and programme staff. Seventeen in-depth interviews and 10 group interviews (total 27) were conducted (Table 1). Review of documentary sources describing Mitanin’s action on social determinants, that mostly included reports and documents of the Mitanin Programme, articles and media reports, and statistics, was undertaken.

**Table 1:** Number of individual and group interviews undertaken in both case studies

Data collection	Manendragarh	Durgkondal	Total
Individual interviews of Mitanins	6	6	12
Individual interviews of Mitanin trainers	1	1	2
Individual interviews of district co-ordinators	1	1	2
Individual interviews of SHRC state-level official			1
Group interviews with Mitanins	1	2	3
Group interviews with community women	2	1	3
Group interviews with Mitanin trainers	1	2	3
Group interviews with block co-ordinators		1	1
Total individual interviews	8	8	17
Total group interviews	4	6	10
Total	12	14	27

The first author (S.N.) undertook data collection with logistical support from members of the Mitanin Programme. All interviews were done in Hindi language and in the specific dialect of the study location and were recorded, transcribed and translated.

Data collection was done in phases and simultaneously with analysis so that the researcher could go back to participants or new participants in order to clarify or verify certain issues or validate the emerging findings and explanatory framework.

### Analysis

In order to facilitate data collection and analysis, a conceptual framework was developed at the start of the research. This ultimately evolved into an explanatory framework (see Figure 2).

The analysis in the multi-case study design is 2-fold (Yin 2009). First, each case was analysed and written up, then the two cases were brought together to draw cross-case conclusions. As with the multiple case-study design, analytical generalization was attempted through replication logic (Yin 2009). The analysis of the data was done following the three processes of description, classification and connection (Gifford undated). At every step, the conceptual framework was revisited and modified accordingly.

One of the limitations of the study was that though at every site interviews were conducted with the community, most of the interviews conducted were of Mitanins and their trainers themselves. This may have resulted in an overstated account of the impact of the programme. However, the community was specifically asked about the impact of the programme and their responses were consistent with what the Mitanins and trainers stated. As an insider to the programme, and especially since she had worked in one of the sites, there was the danger of researcher’s experience influencing the analysis. Remaining conscious of this in data collection and analysis, and the involvement of an independent outsider (H.S.) in the research process, enabled some degree of reflexivity and distance.

## Results

### The Mitanin Programme in the blocks

**Figure 1:**
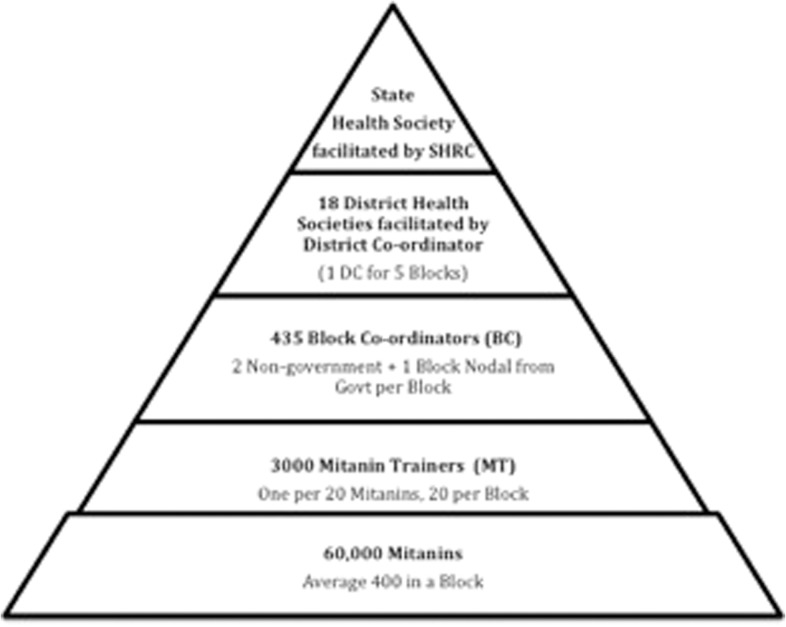
Mitanin programme structure.

The Mitanin Programme structure in all blocks is similar and shown diagrammatically in Figure 1. Although funds flow through the government institutions, all the people in the support structure are contractual employees, recruited from civil society. Mitanin training is conducted through a training cascade from the state level to the Mitanins.

The Mitanin Programme started in Manendragarh in 2002 during the first phase of the programme whereas in Durgkondal it was initiated in the second phase in 2003. In both blocks, the first step was for the block health officials to select the block co-ordinators. The block co-ordinators and the Auxiliary Nurse and Midwife (ANM) in turn selected women mobilizers who later became Mitanin trainers. The mobilizers conducted meetings in the villages and facilitated selection of Mitanins in consultation with the village councils. Folk media was also used for social mobilization around the programme. In both Durgkondal and Manendragarh, a large number of Mitanin trainers and block co-ordinators had been Mitanins previously.

In order to strengthen the Mitanin’s work on nutrition issues, SHRC introduced a Nutrition Fellow in Durgkondal block to focus on nutrition issues, whereas in Manendragarh block, the Mitanin Programme was complemented by an intervention called the Koriya Initiative that was started around the same time by one of the collaborators of the Mitanin Programme. This initiative aimed to mobilize indigenous communities to fight for their rights (SHRC State official Interview).

## Situation of the social determinants before Mitanins started working on them

In Durgkondal block, the prevailing issues included a combination of poor functioning of food programmes and traditional practices related to denial of food to women and children. Various programmes such as mid day meals in schools, Integrated Child Development Services (ICDS) a pre-school nutrition programme and Public Distribution System (providing subsidized grain to poor) were not functioning as intended, and access of the community to these schemes was limited.

In the feeding programme, earlier they were not providing proper food and gave food of bad quality. *(Mitanin group interview, Durgkondal*)

They [community] did not give food for 2–3 days after delivery. They would not give lactating women rice, or green vegetables but only rice water and that too once a day. *(Mitanin group interview, Durgkondal)*

In Manendragarh, deeply entrenched norms of gender inequity meant that women were frequently subjected to various forms of violence, including physical violence, sexual/physical abuse, the withholding of food and verbal abuse. Respondents described how men would be free to act in any way they wanted and were unafraid to inflict violence. Women, on the other hand, felt they could not ask their families, neighbours or the village for help, who instead would find fault with her. Police too were described as non-responsive and as inflicting secondary victimization by harassing and humiliating women complaining of violence. The options available to women were either to endure the violence silently or find refuge in their parents’ house.

In both case study sites, the respondents emphasized the lack of awareness of and information on rights and entitlements. This prevented them from taking any action, resulting in a status quo in the existing situation.

We also did not know how much children are supposed to get. *(Mitanin group interview, Durgkondal)*

We did not have rights and so we would not tell anyone about violence against us. *(community group interview*, *Manendragarh)*

Women also spoke about not being allowed to attend village meetings and of being alienated from any decision-making process within the community.

If we went to meetings we would be asked, why have you come here? There is no role for women in these meetings. *(community group interview, Manendragarh)*

There was a lack of support from and even ridiculing by village councils and administrative structures. The village leaders and elite would question the legitimacy of the Mitanin’s work and would confront her when she went for village meetings.

[They said] so you think you’re great because you have become a Mitanin; what all rubbish meeting you call women for. *(Mitanin trainer group interview, Manendragarh)*

## Process of change

Although different social determinants were studied, the process of taking action was very similar in both cases. The first step was opening up conversations about the issue and building awareness and understanding among local actors—the community, service providers and related institutions. Through hamlet- and cluster-level meetings mostly of women, the Mitanins and Mitanin trainers would share information and discuss issues related to nutrition and food schemes, or gender, women’s health and violence against women. The process involved persistent encouragement to women to act collectively:

Women used to say, why should we go anywhere … We said that … this is also about everyone’s rights—everyone’s children go to feeding center so we all need to act. Slowly, slowly they understood. Not all at once, but one by one. *(Mitanin group interview, Durgkondal)*

We’d tell other women if you don’t help now, how can you expect others to help you when you are in trouble? *(Mitanin trainer interview, Manendragarh)*

Awareness about one’s rights and entitlements led to an increase in use of the services and programmes and enabled the community, especially women, to seek help. In the attempt to change traditional practices related to denial of food to women and children, the Mitanins would counsel the whole family. The Mitanins were able to mobilize the community to intervene in an issue or demand rights and entitlements, and also lead any such action.

Earlier they [ICDS worker and cook] would just cook anything and feed them. I went with village women and made her understand. She then started giving properly. *(Mitanin group interview, Durgkondal)*

Now we have got information about this (violence against women) issue and so we ask for help. *(community group interview, Manendragarh)*

We ensure the woman gets to eat food immediately after delivery … In case the family is telling lies, we sometimes cook food ourselves and feed the woman in front of us. *(Mitanin group interview, Durgkondal)*

The Mitanins would also use the training modules and resource materials as a source of authoritative information in engagement with the service providers and members of the village councils, and thereby created a pressure on them to recognize rights of the community and perform their duties.

We told [mid day meal providers] in the village about menu to be given to the children and showed the book so they had to believe us. *(Mitanin group interview, Durgkondal)*

The Mitanins were also able to elicit help from the Mitanin support structure and also from external persons/agencies such as ICDS worker, supportive government staff, especially when they were unable to resolve an issue.

Mitanins also successfully used local governance structures and forced them to intervene in issues. Issues not resolved at the village or village council level would then be brought up before the government administrative structures and police. Submitting written complaints or applications to block/district officials, in case the situation did not improve through negotiations and pressures at the village level, was a usual practice. In case of violence against women, the Mitanin accompanied the victim to the police to ensure that they took appropriate action and did not further victimize the woman.

The police harass people who are not able to speak up. I have helped many cases in the police station … One should face up to the police also … they are after all humans, not god or anything. They can’t be allowed to be so arbitrary. *(Mitanin trainer interview, Manendragarh)*

Relentless action on any issue and regular follow-ups seem to have ensured success in most interventions that Mitanins took up. They also drew strength from a supportive network, other initiatives and larger campaigns.

We take action on things. We don’t let issues remain. We keep trying till we succeed. *(Mitanin trainer group interview, Durgkondal)*

## Catalysts for action

### Definition of health and role of the Mitanin in the programme

The Mitanin Programme was formulated within the framework of PHC and aimed to address health inequity through action both on the health system and social determinants (SHRC 2003a). Mitanins were envisaged as ‘activists’, who would address issues of the poor and marginalized and lead the social process of their empowerment (SHRC 2003a). The concept of health was defined in a comprehensive manner, encompassing social determinants such as gender, violence against women, nutrition, health equity and broader social rights and entitlements. This definition, along with the role visualized for the Mitanins was followed through into all aspects of the programme design.

### Selection

The Mitanins’ selection process in both blocks was similar and done through a series of meetings with the community and village councils. The community while selecting took considerations such as mobility, ability to speak, leadership qualities, availability of time and family obligations, along with her being from the same village and of the same socioeconomic profile, into account. It is pertinent that in the selection process, many women who already had a desire to work for the community were chosen as Mitanins. These dimensions enabled Mitanins to identify and intervene in the most critical aspects of their lives and kept them motivated to work on these issues that brought a positive change in their own lives too.

I was always the fighting type. One day I saw a man beating his wife. I went and gave him a couple of slaps. *(block co-ordinator group interview, Durgkondal)*

Because we too have experienced such things ourselves and we understand the pain. (*Mitanin interview, Manendragarh)*

Earlier I would also be beaten but since I became a Mitanin, he does not hit me. I think I will NEVER [emphasis by respondent] leave this work. *(Mitanin interview, Manendragarh)*

### Training and ongoing support

From both case studies it has emerged that training and on-the-job support greatly facilitated the Mitanin’s work on the social determinants. In Durgkondal, the Mitanins started working on nutrition issues after the trainings on child health and nutrition and on social security programmes (SHRC 2003b, 2006). Mitanin’s work on violence against women in Manendragarh started after receiving training on it in the women’s health module (SHRC 2003c).

[We work on nutrition] because we were trained for this and told in training to do this. We learnt it from the book. *(Mitanin group interview, Durgkondal)*

The training design built perspective, skills and knowledge about the issue and ways to work on it. It also gave the Mitanins the motivation, confidence and legitimacy to work on social determinants of health.

If every day we are getting beaten, [then] how will we stay healthy? Mentally also don’t stay well … don’t eat well. If someone is physically ill, then we can give medicine, but in this case we have to act and fight against it and only then can one get well. *(Mitanin trainer group interview, Manendragarh)*

When we got training and we were told to go and see these things and improve the situation, we then got to know that we have the right to intervene in these matters too. *(Mitanin group interview, Durgkondal)*

The system of on-the-job training through meetings and field-level support and at times direct action by the Mitanin support structure has been instrumental in sustaining action on social determinants.

### Significance of the Mitanin’s work to the community

Providing health services to the community, especially to women and children played a critical part in securing the community’s trust and confidence in the Mitanins, facilitating their work on social determinants. This included ensuring immunization, giving primary treatment, referring patients to hospitals, promoting delivery in health facilities and taking care of pregnant women and neonates.

They have also played a role in ensuring regular attendance of doctors in health facilities, reducing malpractices by health workers and limiting out of pocket expenditure during delivery in the government hospitals.

Through these various processes, they effectively created a social safety net for women and children:

The villagers think that we have made the mother and child secure. *(Mitanin group interview, Durgkondal)*

The Manendragarh case study shows that the Mitanin’s successful efforts on nutrition and forests helped them to build an organization to take action on any social determinant (Nandi 2005; Dasgupta 2012).

We have understood our power and strength and so have the villagers. *(community group interview, Manendragarh)*

### Community accountability and payment

At all stages of the programme, a conscious emphasis was given to the involvement of the village councils even though the democratic functioning of these councils is of concern (SHRC 2003a). This also helped in fixing accountability of the Mitanins to the community rather than the health department, thereby giving her autonomy from the latter.

The Mitanins are volunteers and do not get any regular remuneration. Although they started receiving certain small incentives with the inception of the ASHA Programme, the rewards were primarily ones of social recognition. Mitanins expressed that they enjoyed meeting other women, people recognizing them and learning new things. Their family members were also happy that they were recognized for their good work. They spoke of being feted by village council members on 23 rd November, the ‘Day of the Mitanin’. Many Mitanins have stood for elections and have been elected to the village councils.

## Environmental factors

The tribal nature of both blocks under study seems to have facilitated the action on social determinants. Such blocks with high indigenous population are marked by impoverishment, marginalization and exploitation and severe issues of gender discrimination, violence and denial of rights (Public Health Resource Network [PHRN] 2010). Moreover, the enabling role of the traditional systems of conflict resolution, as in the case of Durgkondal and the relative openness in society, as in the case of Manendragarh emerged from the case studies.

Initiatives, such as the Nutrition Fellow initiative in Durgkondal and the initiative on tribal rights in Manendragarh and exposure to larger campaigns on food rights and gender rights, have also strengthened the Mitanin’s work in these blocks.

## Challenges faced

In both cases, the Mitanins and the women were often subject to harassment by the village elites or by government workers who faced challenges to their power, privilege and even economic advantage.

A specific threat to the Mitanin’s work on social determinants that emerged was of undue pressure by government workers on Mitanins to fulfil their healthcare targets. This, along with demand for government employment for Mitanin by certain groups, threatened to discourage any action on social determinants, by way of over-medicalization of the programme and reduced autonomy of the Mitanins.

## Roles that emerged for the Mitanins

Mitanins fight for everything, help in everything. *(Mitanin trainer group interview, Manendragarh)*

Both case studies show that the Mitanins worked on a range of issues. They were engaged in healthcare work, which included caring for pregnant and lactating women, neonates and children, providing primary treatment, and motivating the community to demand and utilize health services and also worked on various social determinants. There seems to emerge two types of roles and interactions by the Mitanins.

### 

#### Role as a change agent within the community

As a change agent within the community, the Mitanins built awareness and understanding within the community on malnutrition, violence against women, entitlements and the role of the service providers, and brought about changes in attitudes and practices against the disadvantaged groups. They also compelled the community to respond collectively to the problems and needs of the disadvantaged groups, akin to what Freire (1996) described as ‘conscientization’.^2^

#### Role as an advocate for the community

The second role of the Mitanins was as an advocate for the community vis-à-vis officials and institutions often extending beyond their villages, such as the police, health service and other government workers. In this role, the Mitanin, along with other villagers or on her own, pressurized or negotiated with a range of formal providers to respond to the people’s problems or provide the entitled service.

The Mitanin was considered by community members as someone knowledgeable, available at all times to help, and who speaks up and acts on all rights, even if her legitimacy was questioned by more powerful people. She was seen as intervening successfully on behalf of the disadvantaged. This corresponded with the self-image of the Mitanins.

## Impact of work

In both cases, interviewees believed there has been a significant improvement in the status of the social determinants and in women’s lives. Women, who previously would have felt helpless, now had a support system, which they could access in case of violence or denial of food. Although the level of empowerment of the Mitanins was much higher than that of other women, nonetheless, many women were able to assert themselves both within their family and in the community. They had been able to bring about some change in the balance of power in favour of women and the poor in the community and village.

Mitanins have made the village safe for women. *(Mitanin interview, Manendragarh)*

Now even a lone woman speaks out. Now they don’t get scared. They have been empowered. *(Mitanin group interview, Durgkondal)*

Women expressed how access to information about their rights and solidarity building had helped them make the shifts. They understood that violence against women was not a personal or family issue but a social one and the Mitanins had been instrumental in bringing about this change in perception. Women now attended village meetings, intervened appropriately and partook in community decision making.

Mitanin Programme has been more advantageous for women than men. We [men and women] sit and discuss as equals. *(community group interview, Manendragarh)*

Earlier women would go to meetings only to listen. Now they go to speak out. *(district co-ordinator interview, Durgkondal)*

The Mitanins were respected and recognized for their work. The village councils and government workers appeared to be more responsive, although a certain amount of antagonism towards the Mitanin and other women remained.

## Discussion

Programmes are ‘theories’ and their vision for change determines the mechanisms or the ‘apparatus’ (Pawson and Tilley 2004). In the Mitanin Programme, health was defined as encompassing social determinants of health and Mitanins envisaged as activists, who would address issues of the poor and marginalized and lead the social process of their empowerment (SHRC 2003a). This vision translated into all aspects of programme design such as selection, training and ongoing support, and systems of accountability and remuneration and ultimately shaped the emerging Mitanin’s role. These appear to be the main catalysts for the Mitanin’s action on social determinants.

From the study, it also emerged that selection by the community (Sanders 1990), with flexible criteria (Krishnamurthy and Zaidi 2005) led to many women having existing leadership qualities and interest in community work being selected through their own reputation or initiative (Schulz *et al.* 2002; Krishnamurthy and Zaidi 2005). State programme officials added that while hamlet-based selection mostly ensured that the Mitanin was of the same socioeconomic profile, the lack of payment made it unattractive for village elites to take up this position (SHRC State official Interview). The Mitanin’s attrition rate of 2.5% (SHRC State official Interview) as against rates of 3.2–77% quoted in literature (Lehmann and Sanders 2007) could also signify the efficacy of the selection procedure, among other facilitative processes.

The findings on the significance of ongoing training, and on-the job support corroborate that of other studies, which found that these are the most critical elements of the programme that build interest, skills, confidence, credibility, legitimacy and leadership (Bender and Pitkin 1987; Schulz *et al.* 2002; Lehmann *et al.* 2004; Krishnamurthy and Zaidi 2005; Ingram *et al.* 2008). The significance of regular meetings (Schulz *et al.* 2002), supportive supervision (van Ginneken *et al.* 2010) and mentoring (Ingram *et al.* 2008) has also been commented upon.

In both studies, certain contextual factors may have contributed to success as the CHW’s work on social determinants has found particular relevance in the marginalized and disadvantaged communities (Farquhar *et al.* 2005; Pérez and Martinez 2008), in instances of political and social injustices (van Ginneken *et al.* 2010) and repression (Labonte 2010). The role of complementary and supportive initiatives highlights the role of civil society networks in supporting the CHW’s work on social determinants (SHRC 2003a).

Structures for accountability and remuneration remain a conflict area among scholars even in the case of more technical CHW roles and their design becomes more critical when addressing social determinants, as they can become facilitating or constraining factors in this action. Many feel that not getting regular payment from government has been one of the factors that have allowed Mitanins to retain their autonomy. The design of the programme sees the health department only in a supportive role and not in a monitoring or supervisory role (SHRC 2003a). In April 2012, the Government decided to place accountability of the Mitanin with the village councils who will make all payments to them (State Health Society, Chhattisgarh 2012). Other support includes a ‘Mitanin Welfare Fund’ for social security measures and reservation for Mitanins as candidates for ANM and Staff Nurse courses.

Scholars suggest that accountability is related to who pays the CHW (Bender and Pitkin 1987) and that unless accountability of the CHW is primarily to the community, especially the poor (Sanders 1990) they will be relegated to being the lowest rung of the health department (van Ginneken *et al.* 2010). Moreover, the role of autonomy and flexibility in work in facilitating the work of CHWs on social determinants has emerged in other research (Werner 1981; Ingram *et al.* 2008; van Ginneken *et al.* 2010).

The fact that the programme was situated within the SHRC, an autonomous institution, rather than the government department, contributed greatly to the way the programme emerged and the roles the Mitanins played with respect to both healthcare and social determinants. However, maintaining this autonomy in the face of increasing institutionalization of programme within the health department is a challenge for SHRC (SHRC State official Interview).

The process of the Mitanin’s action on social determinants was found to be similar in both case studies. Action on social determinants by Mitanins followed processes of raising awareness and mobilizing collectives of women, engaging and revitalizing local political structures, and social action within the community and vis-à-vis the various government service providers. The few researchers who have described the process of action on social determinants by the CHWs suggest processes such as awareness building, uniting and mobilizing people, forming groups, developing priorities, influencing decisions, collaboration and intervening (Schulz *et al.* 2002; Ingram *et al.* 2008).

Two roles emerge for the Mitanins: as a change agent within the community and as an advocate for the community; intervening both in healthcare services and social determinants. The role of CHWs in educating, and empowering the community has been considered critical in work on social determinants (Werner 1981; Becker *et al.* 2004; Wallerstein 2006; Prasad and Muraleedharan 2007; Labonte 2010). Scholars have also found CHWs playing the role of an advocate for the community (Ingram *et al.* 2008; Pérez and Martinez 2008; van Ginneken *et al.* 2010). Pérez and Martinez (2008) found that CHWs are the best persons to understand the community’s needs and deal with the most essential services and rights.

Successful work on healthcare helped the Mitanin to establish her legitimacy. As Sanders (1985, quoted in Lehmann *et al.* 2004, p. 11–12) argues, ‘equipping VHWs with curative skills does not simply provide healthcare to more people, more quickly and more cheaply, but it also gives the VHW greater credibility in the eyes of the community.’ The various outcomes of the Mitanin Programme both in the field of healthcare and social determinants have been documented through articles, reports and independent survey data (Nandi 2005; Batiwalla 2007; Sundararaman 2007; Rajshekhar 2010; EUSPP 2011; Dasgupta 2012).

The challenges faced by the Mitanins have been seen in other programmes too. The CSDH (2007) document states that action on social determinants of health is a political process and will be contentious. Over-medicalization of CHW programmes can result in isolating the CHWs from the community (Sanders 1990; Lehmann *et al.* 2004; van Ginneken *et al.* 2010). It remains a constant struggle for SHRC to institutionalize the programme within the community rather than the government. This is exacerbated by attempts being made to unionize the Mitanins for government employment.

Being qualitative in nature, this study was not be able to give any estimates on the proportion of CHWs addressing social determinants, nor the extent to which the two cases represent the norm or instances of positive deviance in the Mitanin Programme. Rather it gives insight into why something happened in a particular context, why some participated and what were the processes involved (Baum 1995). A large number of cases, including one with an adverse outcome and interviews of health service providers may have lent more dimensions to the research topic at hand and strengthened the development of the conceptual framework.

However, the Mitanin Programme Monitoring Information System and Mitanin Evaluation have shown that action on the social determinants by the Mitanins is happening statewide (EUSPP 2011; SHRC 2011).

## Conclusions

**Figure 2:**
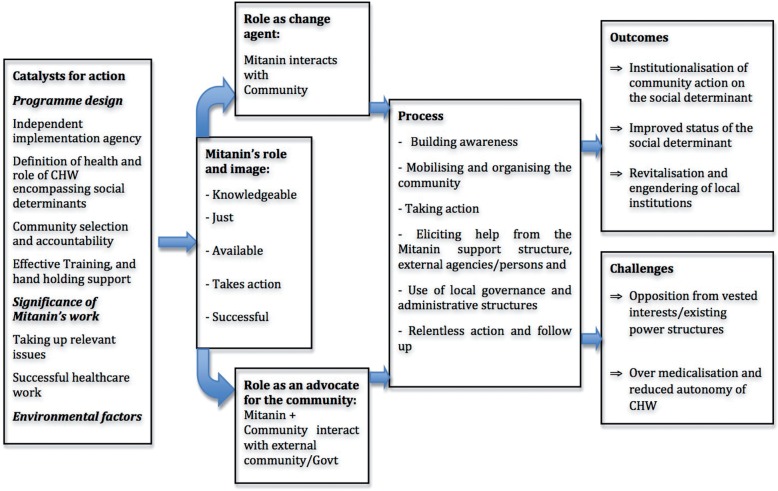
Explanatory framework.

The two case studies, apparently successful examples of action on nutrition- and gender-based violence, have thrown up factors, roles and processes in the action on social determinants. This is illustrated in Figure 2, an explanatory framework for analytical generalization: raising general lessons for programmes in similar contexts. Further research on the Mitanin and the ASHA Programmes, both qualitative and quantitative, should be undertaken, exploring the various emerging themes.

## Ethical clearance

Ethical clearance was obtained from the University of the Western Cape’s Senate Research Committee and permission obtained to undertake the study from the State Health Resource Centre which is the body coordinating the Mitanin Programme in Chhattisgarh. Processes of informed consent preceded all interviews and discussions. After every interview or group discussion there was a debriefing session to allow participants to ask questions and give any further views about the data collection.

## Endnotes

^1^ See, for example, the case of Rambai, a Mitanin living in a hamlet in the forested area of Manendragarh block (one of the case study sites), who not only actively promoted maternal child health but also tackled the case of a drunken school teacher (having him removed from the local school) and successfully led a mobilization to prevent illegal logging in the local forests (www.hindustantimes.com/news-feed/chunk-ht-ui-indiaawakened-topstories/the-mothering-effect/article1-830744.aspx).

^2^ ‘The term “conscientization” refers to learning to perceive social, political and economic contradictions, and to take action against the oppressive elements of reality-Translator’s note’ (Freire 1996, p. 17).
